# A Sequence of Flushing and Drying of Breeding Habitats of *Aedes aegypti* (*L*.) Prior to the Low Dengue Season in Singapore

**DOI:** 10.1371/journal.pntd.0004842

**Published:** 2016-07-26

**Authors:** Osama M. E. Seidahmed, Elfatih A. B. Eltahir

**Affiliations:** Ralph M Parsons Laboratory, Massachusetts Institute of Technology, Cambridge, Massachusetts, United States of America; Mahidol University, THAILAND

## Abstract

In dengue-endemic areas, transmission shows both a seasonal and interannual variability. To investigate how rainfall impacts dengue seasonality in Singapore, we carried out a longitudinal survey in the Geylang neighborhood from August 2014 to August 2015. The survey comprised of twice-weekly random inspections to outdoor breeding habitats and continuous monitoring for positive ones. In addition, observations of rainstorms were collected. Out of 6824 inspected habitats, 67 contained *Aedes aegypti*, 11 contained *Aedes albopictus* and 24 contained *Culex spp*. The main outdoors habitat of *Aedes aegypti* was storm drains (54/67). We found that 80% of breeding sites in drains (43/54) were lost after intense rainstorms related to the wet phase of the Northeast monsoon (NE) between November 2014 and early January 2015. Subsequently, 95% (41/43) of these flushed drains had dried out during the dry phase of the NE in late January-February 2015. A return in the outdoor breeding of *Aedes aegypti* was observed after the onset of Southwest monsoon (SW) between May and August 2015. There was also a reduction in productivity of breeding habitats for larvae and pupae after the onset of the NE. In wet equatorial regions like Singapore, rainfall varies with the monsoons. A monsoon-driven sequence of flushing and drying shapes the outdoor seasonal abundance of *Aedes aegypti*. This finding can be used to optimize vector control strategies and better understand dengue in the context of climate change.

## Author Summary

Increasing concerns about the arboviral diseases transmitted by *Aedes aegypti* mosquito—which include dengue, Chikungunya, yellow fever and Zika virus—demand a better understanding for the breeding ecology of this mosquito. A common observation on mosquito-borne diseases in endemic countries is that they peak following the onset of the rainy season and increase in breeding habitats, while they trough in the dry season. On the contrary, in Singapore, which has no pronounced dry season, dengue cases decrease after a very wet monsoon. Here, we show that this monsoon is likely involved in a strong seasonal reduction of outdoor breeding of the dengue mosquito through a sequence of flushing and drying events. A flushing-drying mechanism may affect the seasonal abundance of the vector in similar eco-epidemiological settings.

## Introduction

Dengue is an increasing public health problem in the world [1]. In endemic countries, dengue transmission shows both a seasonal and interannual variability [2,3]. Although there are various climatic and non-climatic factors that underlie temporal variability, seasonal patterns of dengue coincide with changes in monsoon systems in the tropics [4–6]. For example, in Mexico and Thailand, dengue incidence increases during their main rainy seasons between June and November [7,8]. Similarly, endemic countries in the southern hemisphere, like Brazil and Indonesia, witness dengue peaks in their rainy seasons between January and May [9,10]. Vector control is shown to be effective against dengue transmission when applied early in the season [3]. In addition, there is growing evidence for vector adaptation to outdoor breeding that can increase impact of the climate change on dengue [11,12]. Understanding the climatic drivers of seasonality can improve not only disease surveillance and control in endemic areas, but also global health efforts since tourists visit endemic countries on seasonal holidays [13].

Several studies on dengue and climate have revealed the pivotal role of temperature on both spread and seasonality of dengue [14–16]. Temperature affects larval behavior and development [17–19], survival and biting rate of the adult mosquito [19–21], and extrinsic incubation of the virus in the mosquito [22,23]. Moreover, daily temperature range (DTR) can influence infective probability of dengue virus (DENV) in *Aedes* females [24].

In wet tropical areas, there is little difference in temperature between the seasons, while rainfall occurs throughout the year and only differs in magnitude between the seasons. Rainfall mainly impacts dengue by generating physical conditions for the breeding of the vector. Rainwater can stagnate into a natural breeding habitat or feed an artificial one where mosquitoes can lay eggs [25,26]. On the other hand, rainfall intensity may have negative effects on larvae by pushing them down the water column or washing them out farther from the breeding site or shortening the survival of adults [27,28].

**Fig 1:**
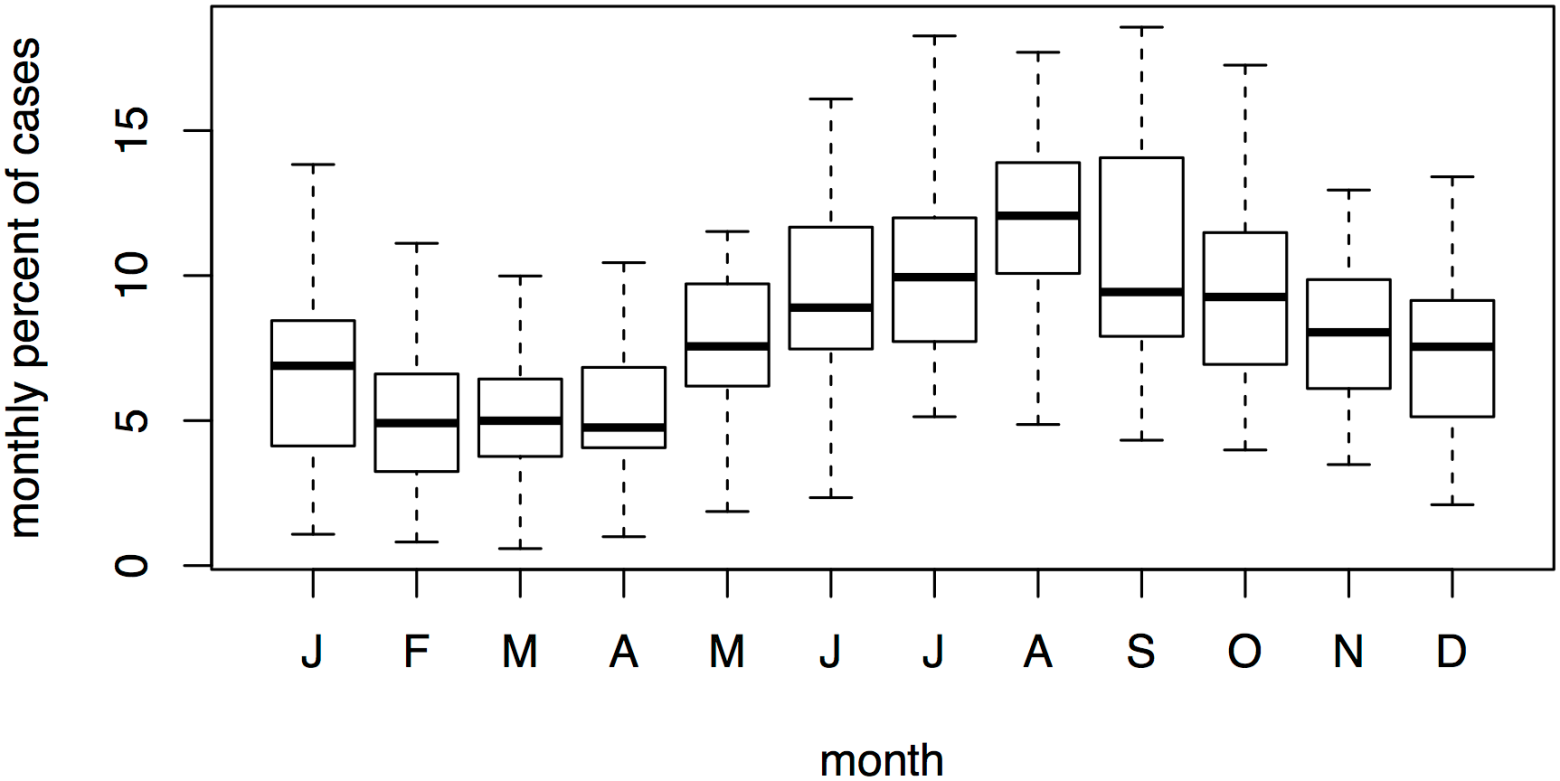
Seasonality of dengue in Singapore: boxplot of monthly percent of cases (1983–2015).

Singapore is a dengue-endemic country where the four serotypes of the virus simultaneously circulate in the city (DENV-1, -2, -3, and -4) [29–32]. While both *Aedes aegypti* and *Aedes albopictus* coexist in Singapore, the latter species is the main disease vector [33]. The city has been struck by repetitive outbreaks during the last two decades. This interannual variability of dengue is attributed to switches in dominant DENV strains and introductions of new virus genotypes [34]. In addition, the disease shows a seasonal peak around July—September and a relatively low incidence in February—April (see Fig 1).

**Fig 2:**
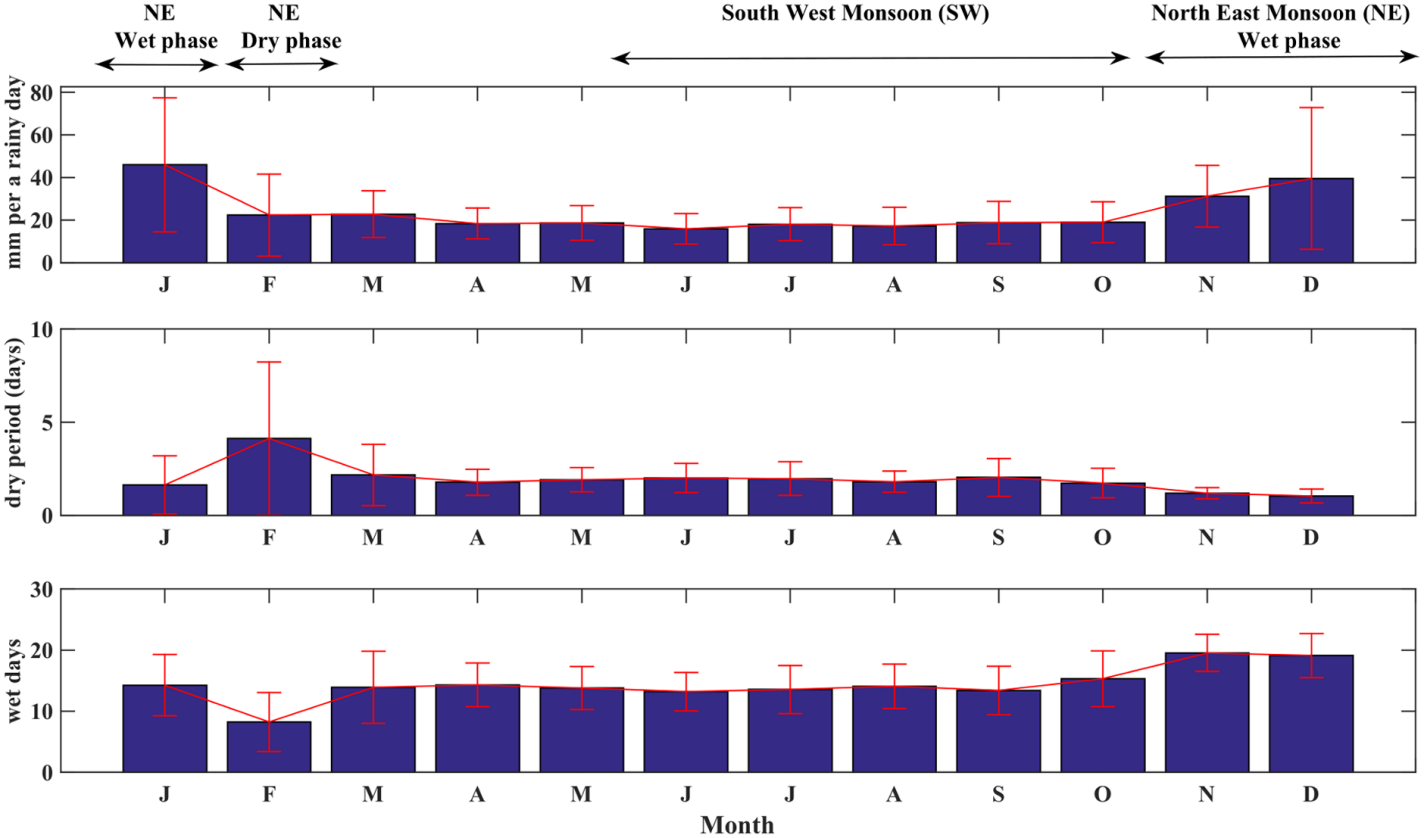
A) Mean daily intensity of rainfall in Singapore per a rainy day (1983–2011). The intensity is calculated by dividing amount of rainstorms by rainy days of a month. B) Mean duration of a dry period (i.e. sum of hourly stretches without rainfall) without a rainstorm. C) Monthly average numbers of rainy days; notice February is the driest month with only eight rainy days. Arrows indicate the Northeast (i.e. the wet and dry phases, NE1 and NE2, respectively) and Southwest (SW) monsoons. Data source: Changi station—National Environmental Agency (NEA).

Singapore is also subject to two monsoons: a Northeast monsoon (NE) that results in heavy rainfall between November and March and a relatively drier Southwest monsoon (SW) between June and October [35]. Interestingly, the seasonal trough of dengue cases follows the NE. Daily rainfall intensities are higher by a magnitude of 12–25 mm during the wet phase of the NE (i.e. November-January) compared to other months (see Fig 2A). In addition, the dry phase of the NE places February as the driest month, where dry periods extend to four days and a total of only eight rainy days (see Fig 2B and 2C, respectively). On the other hand, hourly temperature in Singapore does not exceed 2°C between the seasons in Singapore. The mean temperature of the hottest and coolest months, May and December, are 28.4°C and 26.5°C, respectively (see S1 Fig).

Past studies have shown statistical relationships between dengue and climate in Singapore. Heng and others showed a rise in weekly temperature 8–20 weeks in advance preceded an increase in dengue incidence in Singapore. This lag time was found to be 18 weeks during the major outbreak of 2005 [33]. Researchers also used weekly mean temperature and cumulative rainfall to identify a 16-week period as the optimum to forecast dengue outbreaks in Singapore [5]. In a recent work, absolute humidity showed strong predictive value for dengue incidence [36].

Here, we provide a mechanistic basis to explain the connection between dengue and rainfall in Singapore. We show that the NE is likely involved in a strong seasonal reduction of outdoor breeding of the dengue mosquito through a sequence of flushing and drying events.

## Methods

### Biosafety and ethics statement

This study received a risk assessment approval from the Institutional Biosafety Committee (IBC) of Singapore-MIT Alliance of Research and Technology (SMART). The research was not conducted in any private residences and no human samples were collected.

### Hypothesis

A preliminary entomological survey was carried out in July 2013 in Singapore. Three neighborhoods were inspected for outdoor breeding of the dengue vector: Geylang (1.320° N, 103.891° E), Lorong Limau (1.323° N, 103.855° E) and Caldecott (1.337° N, 103.839°E). Accordingly, we found that roadside drains in back alleys are the main outdoor breeding habitats of *Ae*. *aegypti*. Breeding was also encountered in discarded receptacles indoors. While *Ae*. *albopictus* was identified in various outdoor discarded receptacles (but in association with the canopy), *Culex spp*. was mainly found in large drains on the main lanes and roads.

**Fig 3:**
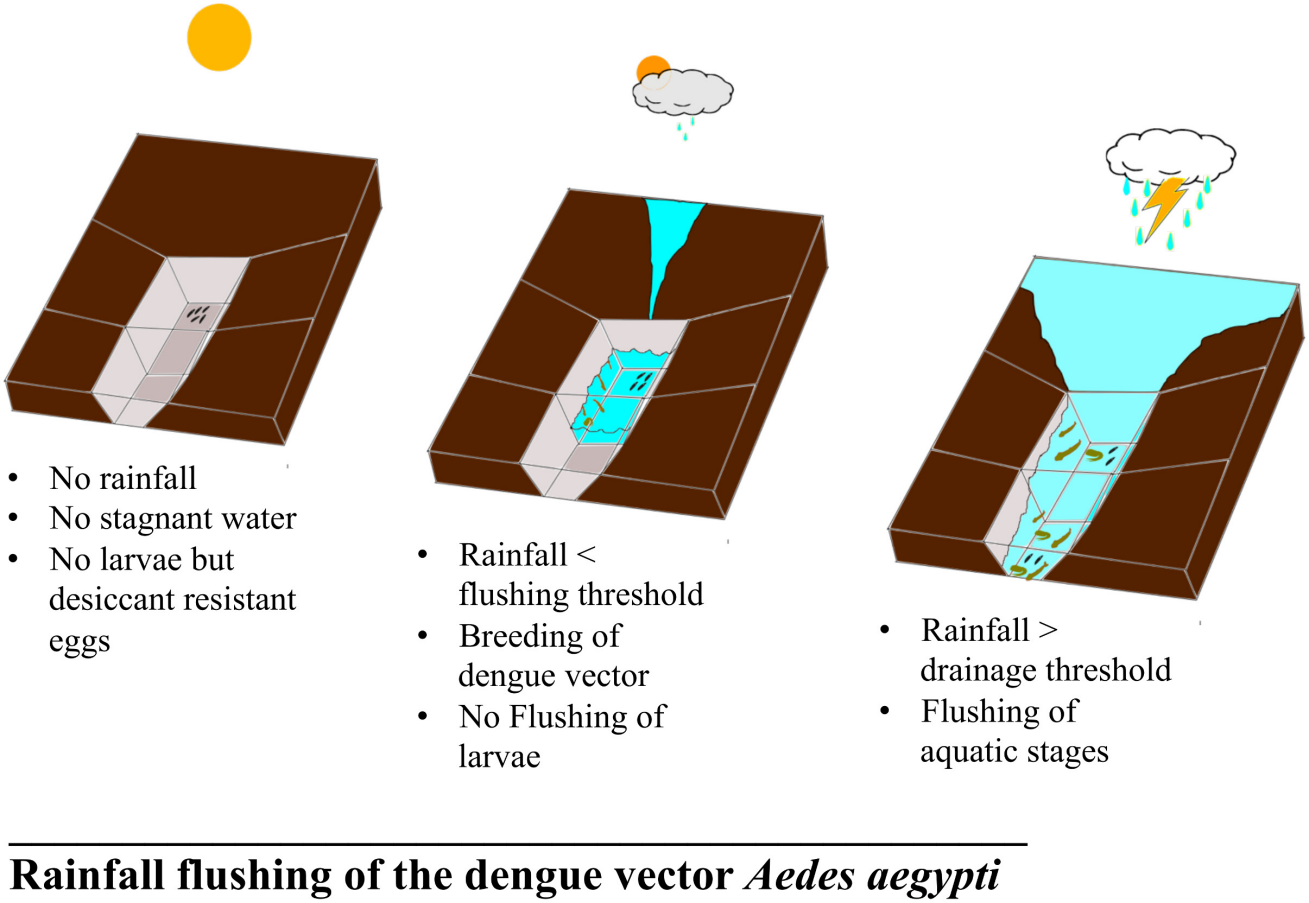
A descriptive sketch for the rainfall flushing mechanism shows how intense rainstorms during the monsoon results in washing breeding of dengue vector from stagnant drains.

Based on the main observation of the preliminary survey (i.e., that dengue vector *Ae*. *aegypti* breeds in drains) combined with the above epidemiological and meteorological findings (see Figs 1 and 2), we developed a hypothesis to explain the connection between rainfall and outdoor breeding of the dengue vector in Singapore. During dry periods, only desiccation-resistant eggs can survive in drains and similar outdoor breeding habitats. We hypothesize that while a monsoon results in breeding of *Ae*. *aegypti* in drains, a monsoon with intense rainstorms can cause flushing of aquatic stages (see Fig 3). In order to test this hypothesis, we selected Geylang as study area.

#### Study area

**Fig 4:**
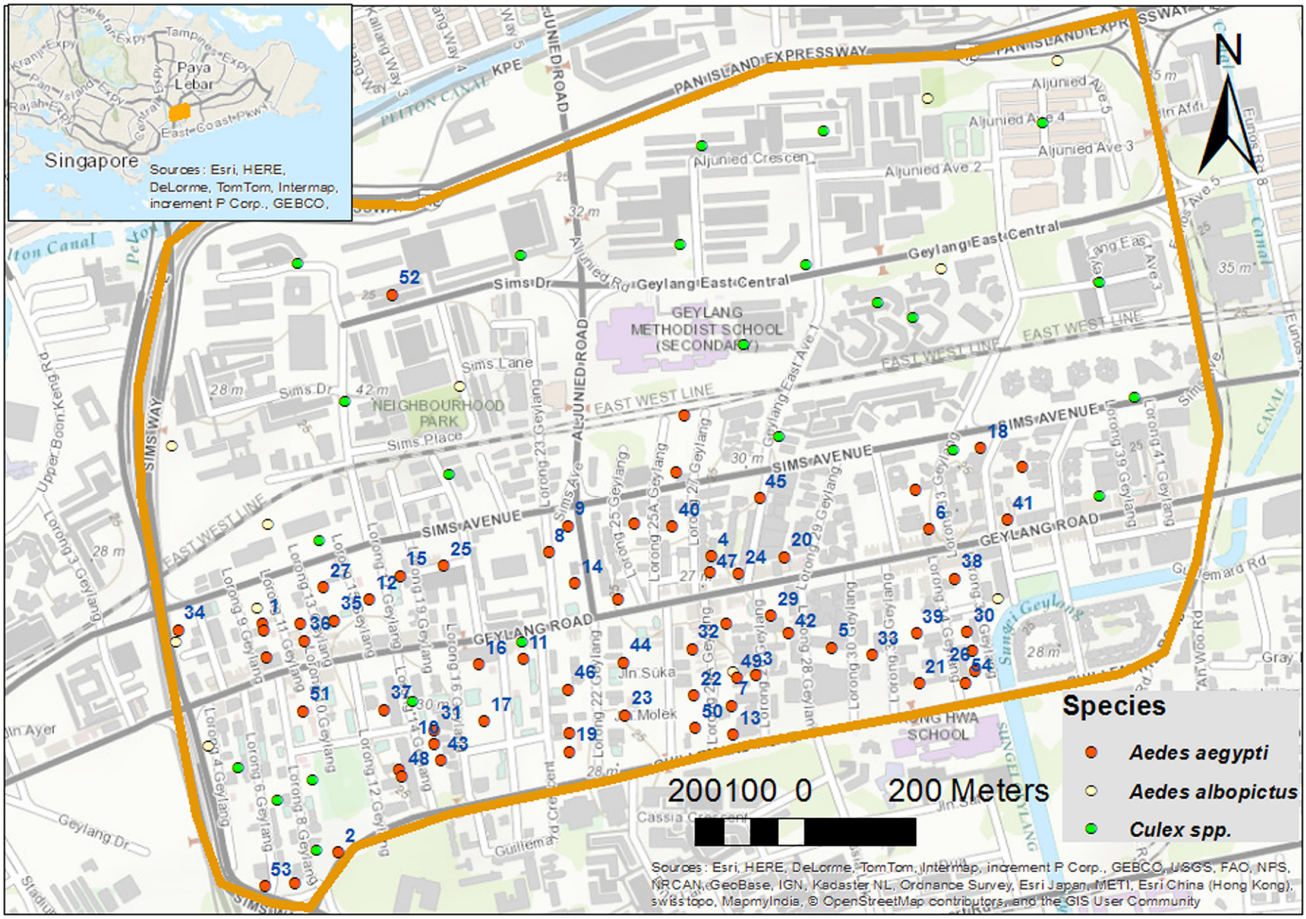
The study area: Geylang neighborhood, Singapore. The figure shows locations of positive breeding sites for *Aedes aegypti*, *Ae albopictus* and *Culex spp*. Breeding drains of *Ae*. *aegypti* are given serial numbers that also used in Fig 6.

Geylang neighborhood, east of the Singapore River, is a highly urbanized neighborhood that has an area of about 3 km^2^. Although Geylang has an estimate of 32,000 residents population, non-residents is believed to be larger because of the cheap housing that attracts foreign laborers. National Environmental Agency (NEA) recognizes Geylang as a hyperendemic area where a continuous reporting of dengue cases and disease transmission happens (see Fig 4).

### Entomological surveys

Entomological surveys were continuous between August 2014 and August 2015 except for two weeks between February 21st and March 10th. The surveys included two tasks of inspections:

#### Semiweekly-random inspections

We carried out a random aquatic survey twice a week. The inspector was equipped with torchlight, sieves, large-mouth pipettes, a white enamel pan and small shell vials. In each survey, the inspector examined all outdoor natural/artificial habitats in the selected blocks for aquatic stages. Samples of pupae and larvae were pipetted in labeled vials with 70% ethanol, and transferred to the laboratory for taxonomy. In addition, a subsample of aquatic specimens was held alive in a netted cup until adult emergence to confirm identification. Taxonomic keys [37–39] were used to identify the preserved larvae and emerged adults. For a positive breeding habitat of mosquitoes, type of habitat and presence of other aquatic insects were recorded. Location of positive habitats was geo-referenced using GPS tools.

#### Monitoring of positive habitats of *Aedes aegypti*

We also carried out semiweekly monitoring of the positive breeding habitats. In particular, we focused here on breeding history of *Aedes aegypti* in the drains. In particular, the aim was to follow-up these positive drains since the starting date when a breeding of *Ae*. *aegypti* was found (in the regular random inspections) and continuously till the end of the survey in August 2015. Hence, we describe four situations in these monitored sites: 1) Stagnant and Positive (SP), 2) Stagnant and Negative (SN), 3) Flushed and Negative (FN), and 4) dry and negative (DN). In addition, in a case of SP, we estimated the number of larvae and pupae in the site using larval dippers. Larval density per breeding habitat was calculated as the total number of larvae of *Ae*. *aegypti* divided by the number of positive breeding sites in the semiweekly monitoring survey. We also determined pupal-productivity of the breeding habitats by summation of numbers of pupae collected from the positive drains and non-drains during the semiweekly survey.

### Microclimatic data on rainfall and flushing

A set of weather HOBO loggers was placed in Geylang between August 2014 and August 2015 to record hourly microclimatic conditions. These included: a rain gauge to record amounts of rainstorms, and water level logger. The siphon rain gauge tipping bucket (TR-525S) was calibrated in the laboratory according to the manufacturer. Next, the rain gauge was placed on the roof of a 7-storey building to prevent obstruction.

To characterize flushing events, we placed HOBO U20L logger in a back alley drain in Geylang. The water level logger records water temperature and absolute pressure every 10 minutes. Software uses absolute pressure, reference water level and density to calculate the water level. The accuracy of the device is 0.1%. In addition, daily rainfall was obtained from the closest NEA weather station for the same period.

Logging of the rain gauge was intermittent because of periodic sensor errors. However, readings from nine months (between 9/15-9/25/2014, 12/07/2014-3/26/2015 and 5/28-8/26/2015) are retrieved. In order to overcome this discrepancy, we compensated missing data by their corresponding rainfall from the closest station Tanjong Katong. A regression analysis has showed that data of the weather station could strongly predict data of the rain gauge (R^2^ = 0.94).

## Results

### Entomological observations

#### Main outdoors breeding habitats

Out of 6824 inspected sites, 3624 (53%) were wet habitats. Most of outdoor breeding habitats were open and closed drains, 45% and 40%, respectively. The remained of inspections (15%) were non-drains including canvas sheets, pails, plastic bags, and flowerpots. Interestingly, positive habitats contained *Ae*. *aegypti* (n = 67), followed by *Culex spp*. (n = 24) and *Ae*. *albopictus* (n = 11)- Table 1. Breeding of *Ae*. *aegypti* was mainly in storm drains (53/67). In addition, we found aquatic stages of non-mosquito species belonging to the families: *Chironomidae*, *Psychodidae* and *Viviparidae*. We also encountered rodents in the drains (Family: *Muridae*).

**Table 1:** Total number of inspected and positive habitats in Geylang August 2014–August 2015.

	Inspected habitats		Positive habitats		
Type of habitat	Dry	Wet	*Ae*. *aegypti*	*Ae*. *albopictus*	*Culex spp*.
**Open drains**	1267	1470	22	2	13
	(46.3%)	(53.7%)	(1.4%)	(0.14%)	(0.88%)
**Closed drains**	1463	1618	31	0	8
	(47.5%)	(52.5%)	(1.9%)		(0.49%)
**Non-drains**	470	536	14	9	3
	(46.7%)	(53.3%)	(2.6%)	(1.68%)	(0.56%)
**Total**	3200	3624	67	11	24
	(46.9%)	(53.1%)	(1.8%)	(0.3%)	(0.6%)

Note: A percentage in a breeding habitat is in reference to the corresponding number of wet habitats.

**Fig 5:**
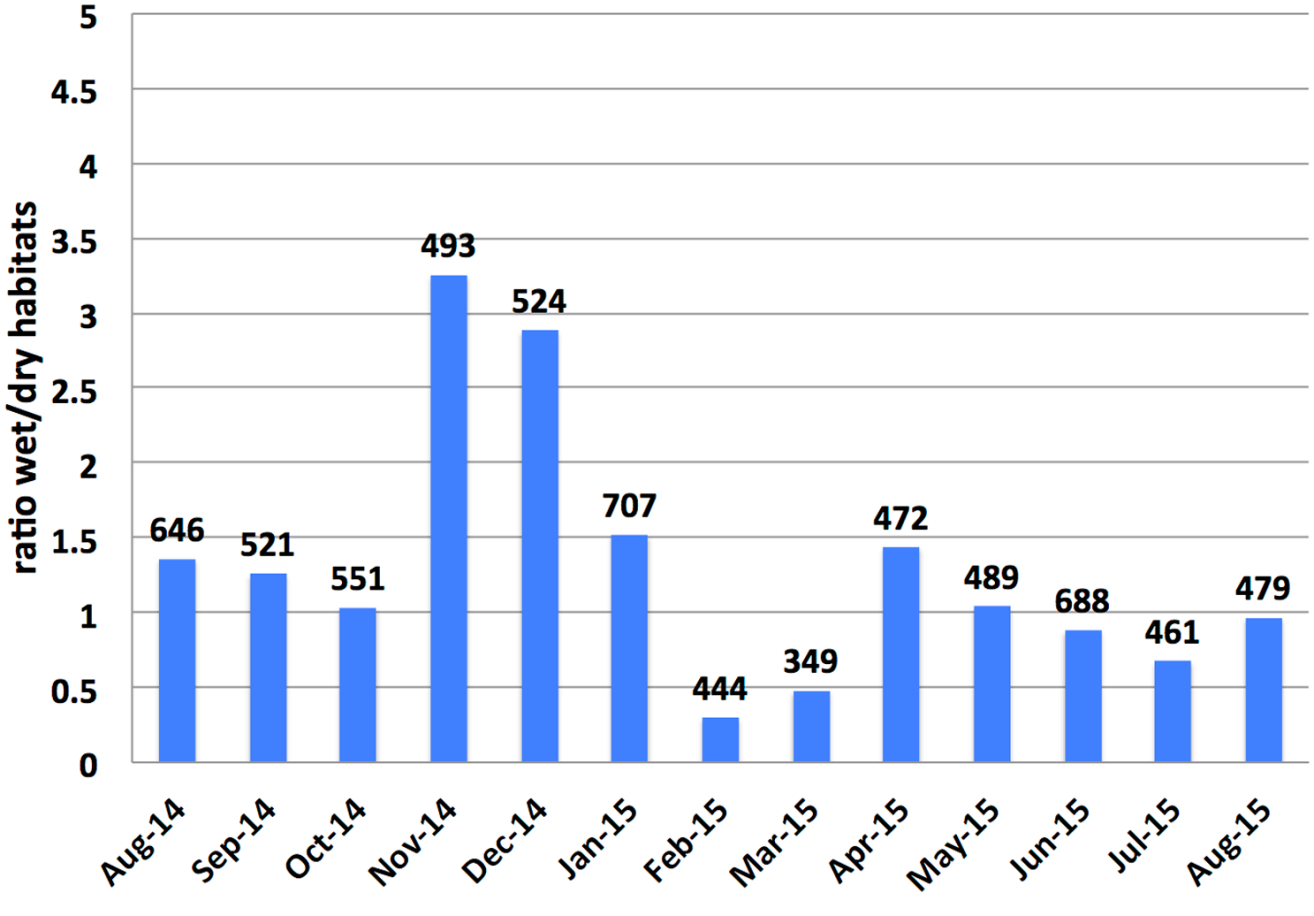
Monthly ratio of wet/dry habitats in Geylang (August 2014–August 2015). Total numbers of inspected habitats are shown above bars. Arrows indicate periods of the wet and dry phases of the Northeast monsoon (NE1 and NE2, respectively) and Southwest monsoon (SW). Note: no inspections were carried out between 2/21/15 and 3/10/15.

The monthly ratio of wet to dry habitats inspected in Geylang is shown in Fig 5. Accordingly, the number of wet habitats found during the wet phase of the NE is more than twice of the dry ones. On the other hand, wet habitats during the dry phase of the NE are less than half of the dry ones.

#### Flushing, drying and return of outdoor breeding

**Fig 6:**
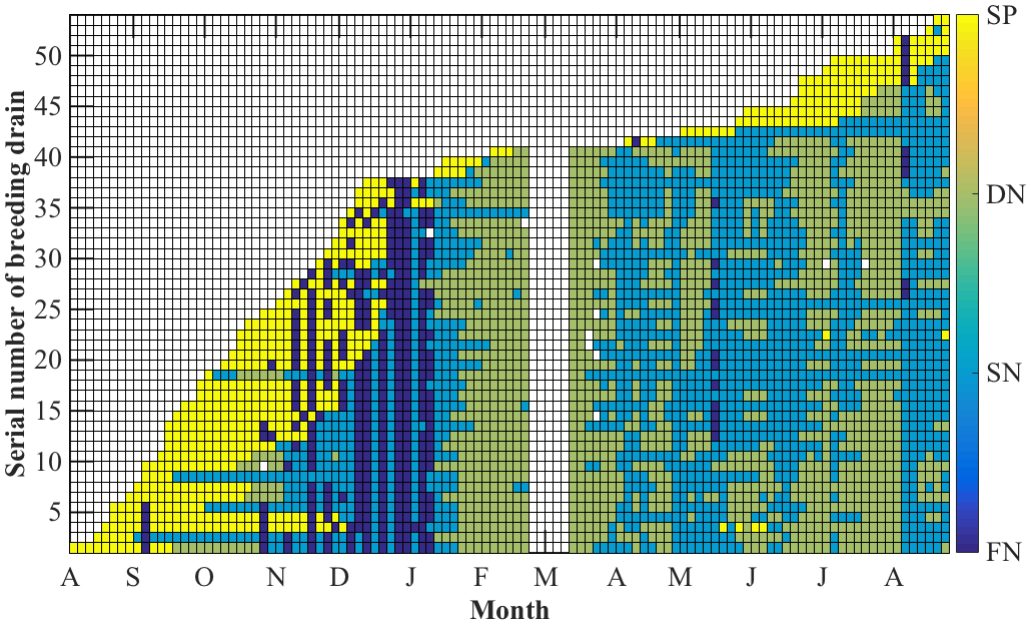
Timeline of the breeding drains of *Aedes aegypti* in Geylang, Singapore: August 2014- August 2015. (SP: stagnant and positive, DN: Dry and Negative, SN: Stagnant and Negative, FN: Flushed and Negative). Grids along the x-axis represent the twice-weekly follow-ups. White grids indicate no inspections were carried out in these drains (two weeks between 2/21/2015 and 3/10/2015). Locations of the breeding drains are shown in Fig 4.

Fig 6 shows that breeding drains were mainly flushed in the wet phase of the NE. Moreover, the following dry phase of the NE had resulted in drying of 95.3% (41/43) of the flushed drains. Monitoring resumed in March showed that most of the previously positive drains were still dry 82.9% (34/41) while the wet ones (7/41) were negative. A return in outdoor breeding was shown after the onset of the SW. Hence, we found 11 positive drains for *Ae*. *aegypti* in June-August 2015. All these drains except one were within 200 meters from the previously positive ones prior to the NE period (see Fig 1).

Locations of the breeding drains are in Fig 4. There is a clustering for breeding drains of *Ae*. *aegypti* in the southern part of Geylang.

#### Effects on aquatic stages of *Ae*. *Aegypti*

**Fig 7:**
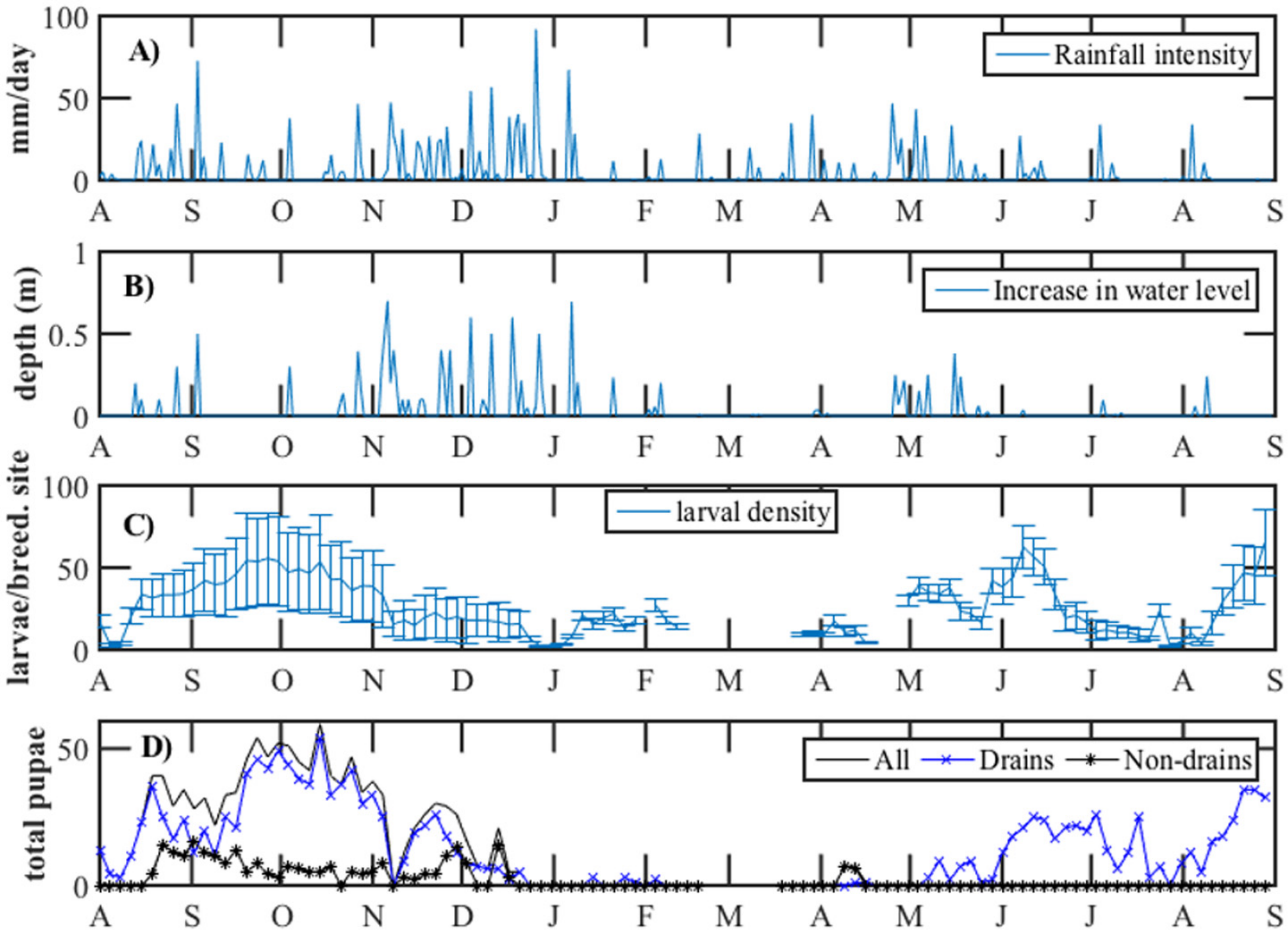
**A.** Intensity of daily rainstorms in Geylang (August 2014–August 2015). **B.** Increase of water level in a drain in Geylang (August 2014–August 2015). **C.** larval density of *Aedes aegypti* per an outdoor breeding habitat in Geylang per semiweekly survey. **D.** Pupal-productivity of drains and non-drains for *Ae*. *aegypti* per semiweekly survey. Note: no monitoring was carried out between 2/21/2015 and 3/10/2015.

Fig 7A shows that 31.2% (19/61) of intense rainstorms (i.e. >10 mm) had occurred during the wet phase of the NE (i.e. November–December 2015). In addition, Fig 7B shows a similar pattern of increases in water level of the monitored drain.

The monsoonal pattern has also influenced larval density per breeding habitat as shown in Fig 7C. These larval densities were higher during the SW periods (averages were 38.1 and 27.7 in 2014 and 2015, respectively) compared to the NE period (average = 16.7).

Similarly, the wetter monsoon had affected pupal-productivity of breeding drains for *Ae*. *aegypti* (see Fig 7D). Pupae had decreased after the onset of the NE from a total of 901 to 284 (i.e. 68.5% reduction). Although few breeding drains were encountered after the onset of the SW in 2015, the total of pupae was double that of the NE period (N = 505).

## Discussion

In Singapore, dengue cases peak during the third quarter of the year while they dip in the first one. For this study, we show rainfall may influence dengue via a sequence of two processes acting on the outdoor population of vector mosquito: 1) intense rainstorms that flush out breeding drains of the main vector, and 2) acute drying that follows and impedes returning of *Aedes aegypti* breeding. While flushing happens when Singapore is under the wet phase of the NE, drying occurs when the monsoon which passes Singapore converges into Inter-tropical convergence zone (ITCZ) over Java [35].

Fluctuations of DTR around monthly mean temperatures 26.5–28.4°C are small in Singapore (i.e., < 1.1°C). Lambrechts and others suggested that small DTR around a mean temperature 26°C could induce the high season of DENV [24]. Hence, we argue that DTR effect on dengue seasonality in Singapore is modest.

We showed that the ratio of wet to dry habitats discovered during our random survey is larger in the wet phase of the NE than in the late dry one. However, wetness is not a sufficient condition for a mosquito to lay its eggs at a specific location. In fact, mosquitoes lay their eggs in specific breeding habitats that minimize mortality risk (e.g., predation or competition) and maximize nutritional benefits for their offspring [40]. One possible explanation for finding new breeding habitats during the wet phase of the NE is that they were flushed from indoor breeding sources. In addition, they can result from hatching of dormant eggs in the drains.

Likewise, we showed the effect of the NE on pupal-productivity and larvae of *Ae aegypti*. The rainfall and water level loggers verified these effects. However, the effect of intense rainstorms could be substantial on larval food; hence, on size of pupae and emerged adults. On the other hand, sampling of pupae from outdoor breeding habitat can be utilized in dengue surveillance in Singapore. Because sampling adults of *Ae*. *aegypti* is difficult, several studies have shown that pupal indices are useful in dengue surveillance [41–43].

A number of factors may explain why few breeding sites found between June and August 2015. First, an intensive larviciding program for the drains is recently introduced in Geylang (observed by the investigators). Second, there was unusual drying in the drains particularly in June-July 2015 that resulted from El Niño. Indeed, there is ongoing strong El Niño in 2015 [44]. The impact of El Niño episodes on dengue in Singapore was previously recorded in Singapore in May-2002-March 2003, June 2004- Feb2005 and Aug 2006-Jan 2007 by Hii and others [30].

We have no information whether breeding in drains had resulted from oviposition at these sites or flushing of indoor or upstream sites. In fact, there were no inspections for indoors habitats due to ethical and legal considerations. For example rain gutters, which are considered by National Environmental Agency of Singapore (NEA) as a key-breeding habitat for *Ae*. *aegypti*, could be the source that inoculated the breeding in drains. There is a need to assess the relative productivity of storm drains—in terms of *Ae*. *aegypti* pupae—to that of other indoor containers. A further work is also needed to determine a flushing threshold that could result in reduction of breeding in drains. This threshold could be an attribute to the drainage network in a neighborhood.

In order to optimize dengue vector control in Singapore and similar wet tropical areas, we suggest seasonal strategies—as in S1 Table. Targets and measures of vector control should consider the difference in outdoor abundance of the vector between pre-seasonal and seasonal periods of the year. A pre-seasonal control strategy should focus on elimination of indoor breeding habitats particularly during the monsoonal dry period. We recommend treatment of breeding drains and roof gutters by long lasting persistence larvicide before the rain arrives and the mosquito flourishes in outdoor habitats. This pre-seasonal strategy can be effective to reduce the disease risk before onset of the high season. Removal of discarded receptacles should be continued around the year. We also propose a focal space spraying—using an adult insecticide—when an outdoor breeding habitat encountered to minimize the dispersal of emerged adults within the flight range of *Ae*. *aegypti*.

There is a growing interest in the health consequences of climate change. While projections of the climate change show an increasing trend in temperature under “the business-as-usual” scenario, the effects are less understood on rainfall distribution and patterns. In equatorial regions, a non-stationary increase in rainfall is expected to follow the seasonal displacement of ITCZ [45]. In general, if climate change enhances the wet conditions around December or enhance the dry conditions around February, then that may impact the seasonality of Dengue in this region. The flushing-drying mechanism may play a role in shaping the impact of climate change on dengue and other related arboviral diseases.

In conclusion, rainfall has a mechanistic role in shaping seasonal abundance of the dengue vector *Ae*. *aegypti* in Singapore. This effect happens through a monsoonal-driven sequence of flushing and drying in outdoor breeding habitats. In light of global urbanization, urban drainage systems are expanding in well-structured urban setting like Singapore. Hence, vector control interventions can be very effective before the dengue season in such eco-epidemiological settings.

## Supporting Information

S1 Fig Variation of the 3-hours average of temperature in Singapore (1983–2011). Data source: Changi station– National Environment Agency of Singapore (NEA).
(TIF)

S1 Table Optimization of dengue vector control strategies according to the monsoon season. (DOCX)

S1 Data Supporting Excel file that contains other relevant data. (XLS)

## References

[1] Bhatt S, Gething PW, Brady OJ, Messina JP, Farlow AW, Moyes CL, et al The global distribution and burden of dengue. Nature [Internet]. Nature Publishing Group; 2013;496(7446):504–7. Available from: http://www.nature.com/doifinder/10.1038/nature12060 10.1038/nature12060 PMC3651993 23563266

[2] Johansson MA, Cummings DAT, Glass GE. Multiyear Climate Variability and Dengue—El Niño Southern Oscillation, Weather, and Dengue Incidence in Puerto Rico, Mexico, and Thailand: A Longitudinal Data Analysis. PLoS Med [Internet]. 2009 11 [cited 2012 Mar 12];6(11):e1000168 Available from: http://dx.plos.org/10.1371/journal.pmed.1000168 10.1371/journal.pmed.1000168 PMC2771282 19918363

[3] Stoddard ST, Wearing HJ, Reiner RC, Morrison AC, Astete H, Vilcarromero S, et al Long-Term and Seasonal Dynamics of Dengue in Iquitos, Peru. PLoS Negl Trop Dis. 2014;8(7):19–21. 10.1371/journal.pntd.0003003 PMC4102451 25033412

[4] Wiwanitkit V. An observation on correlation between rainfall and the prevalence of clinical cases of dengue in Thailand. J Vector Borne Dis [Internet]. 2006 [cited 2015 Nov 12];43(2):73–6. Available from: http://www.mrcindia.org/journal/issues/432073.PDF 16967819

[5] Hii YYL, Zhu H, Ng N, Ng LCL, Rocklöv J. Forecast of Dengue Incidence Using Temperature and Rainfall. PLoS Negl Trop Dis [Internet]. 2012 [cited 2015 Nov 12];6(11):e1908 Available from: 10.1371/journal.pntd.0001908\nhttp://www.plosntds.org/article/fetchObjectAttachment.action?uri=info:doi/10.1371/journal.pntd.0001908&representation=PDF PMC3510154 23209852

[6] Wai KT, Arunachalam N, Tana S, Espino F, Kittayapong P, Abeyewickreme W, et al Estimating dengue vector abundance in the wet and dry season: implications for targeted vector control in urban and peri-urban Asia. Pathog Glob Health [Internet]. 2012;106(8):436–45. Available from: http://www.scopus.com/inward/record.url?eid=2-s2.0-84871949303&partnerID=tZOtx3y1 10.1179/2047773212Y.0000000063 PMC3541889 23318235

[7] Wichmann O, Yoon I-K, Vong S, Limkittikul K, Gibbons R V., Mammen MP, et al Dengue in Thailand and Cambodia: An Assessment of the Degree of Underrecognized Disease Burden Based on Reported Cases. PLoS Negl Trop Dis [Internet]. 2011;5(3):e996 Available from: http://dx.plos.org/10.1371/journal.pntd.0000996 10.1371/journal.pntd.0000996 PMC3066139 21468308

[8] Colón-González FJ, Lake IR, Bentham G. Climate variability and dengue fever in warm and humid Mexico. Am J Trop Med Hyg [Internet]. 2011 5 5 [cited 2016 Jan 19];84(5):757–63. Available from: http://www.ajtmh.org/cgi/content/long/84/5/757 10.4269/ajtmh.2011.10-0609 PMC3083744 21540386

[9] Siqueira JB, Martelli CMT, Coelho GE, Simplicio ACDR, Hatch DL. Dengue and dengue hemorrhagic fever, Brazil, 1981–2002. Emerg Infect Dis [Internet]. 2005 [cited 2015 Nov 4];11(1):48–53. Available from: http://www.ncbi.nlm.nih.gov/pmc/articles/PMC3294356/10.3201/eid1101.031091 PMC3294356 15705322

[10] Arcari P, Tapper N, Pfueller S. Regional variability in relationships between climate and dengue/DHF in Indonesia. Singap J Trop Geogr. 2007;28(3):251–72.

[11] Chadee DD, Martinez R. Aedes aegypti (L.) in Latin American and Caribbean region: With growing evidence for vector adaptation to climate change? Acta Trop [Internet]. Elsevier B.V.; 2016;156:137–43. Available from: http://linkinghub.elsevier.com/retrieve/pii/S0001706X1530200X 10.1016/j.actatropica.2015.12.022 26796862

[12] Manrique-Saide P, Uc V, Prado C, Carmona C, Vadillo J, Chan R, et al Storm Sewers as Larval Habitats for Aedes aegypti and Culex Spp. in a Neighborhood of Merida, Mexico. J Am Mosq Control Assoc. 2012;28(3):255–7. 23833907 10.2987/12-6244R.1

[13] Wilder-Smith A. Dengue infections in travellers. Paediatr Int Child Health [Internet]. 2012 5;32 Suppl 1:28–32. Available from: http://www.pubmedcentral.nih.gov/articlerender.fcgi?artid=3381444&tool=pmcentrez&rendertype=abstract 10.1179/2046904712Z.00000000050 PMC3381444 22668447

[14] Morin CW, Comrie AC, Ernst K. Climate and dengue transmission: Evidence and implications. Environ Health Perspect. 2013;121(11–12):1264–72. 10.1289/ehp.1306556 24058050 PMC3855512

[15] Hopp MJ, Foley JA. Global-scale relationships between climate and the dengue fever vector, AEDES AEGYPTI. Clim Change. 2001;48(2–3):441–63.

[16] Eisen L, Monaghan AJ, Lozano-Fuentes S, Steinhoff DF, Hayden MH, Bieringer PE. The impact of temperature on the bionomics of Aedes (Stegomyia) aegypti, with special reference to the cool geographic range margins. J Med Entomol [Internet]. 2014;51(3):496–516. Available from: http://www.ncbi.nlm.nih.gov/pubmed/24897844 10.1603/me13214 24897844

[17] Focks DA, Haile DG, Daniels E, Mount GA. Dynamic life table model for Aedes aegypti (Diptera: Culicidae): analysis of the literature and model development. J Med Entomol [Internet]. 1993 11;30(6):1003–17. Available from: http://www.ncbi.nlm.nih.gov/entrez/query.fcgi?db=pubmed&cmd=Retrieve&dopt=AbstractPlus&list_uids=8271243\nhttp://www.ncbi.nlm.nih.gov/entrez/query.fcgi?db=pubmed&cmd=Retrieve&dopt=AbstractPlus&list_uids=8271242 10.1093/jmedent/30.6.1003 8271242

[18] Reiskind MH, Janairo MS. Late-instar Behavior of *Aedes aegypti* (Diptera: Culicidae) Larvae in Different Thermal and Nutritive Environments. J Med Entomol [Internet]. 2015 9;52(5):789–96. Available from: http://jme.oxfordjournals.org/lookup/doi/10.1093/jme/tjv088 10.1093/jme/tjv088 26336228

[19] Delatte H, Gimonneau G, Triboire a, Fontenille D. Influence of temperature on immature development, survival, longevity, fecundity, and gonotrophic cycles of Aedes albopictus, vector of chikungunya and dengue in the Indian Ocean. J Med Entomol. 2009 1;46(1):33–41. 19198515 10.1603/033.046.0105

[20] Carrington LB, Armijos MV, Lambrechts L, Barker CM, Scott TW. Effects of Fluctuating Daily Temperatures at Critical Thermal Extremes on Aedes aegypti Life-History Traits. PLoS One [Internet]. 2013;8(3):e58824 Available from: http://dx.plos.org/10.1371/journal.pone.0058824 10.1371/journal.pone.0058824 PMC3592833 23520534

[21] Halstead SB. Dengue Virus–Mosquito Interactions. Annu Rev Entomol [Internet]. 2008;53(1):273–91. Available from: http://www.annualreviews.org/doi/abs/10.1146/annurev.ento.53.103106.093326 10.1146/annurev.ento.53.103106.093326 17803458

[22] Focks D a., Brenner RJ, Hayes J, Daniels E. Transmission thresholds for dengue in terms of Aedes aegypti pupae per person with discussion of their utility in source reduction efforts. Am J Trop Med Hyg [Internet]. 2000 1;62(1):11–8. Available from: http://www.ncbi.nlm.nih.gov/pubmed/10761719 10761719

[23] YANG HM, MACORIS MLG, GALVANI KC, ANDRIGHETTI MTM, WANDERLEY DM V. Assessing the effects of temperature on the population of Aedes aegypti, the vector of dengue. Epidemiol Infect [Internet]. 2009 8 [cited 2012 Jul 24];137(08):1188 Available from: http://www.journals.cambridge.org/abstract_S0950268809002040 10.1017/S0950268809002040 19192322

[24] Lambrechts L, Paaijmans KP, Fansiri T, Carrington LB, Kramer LD, Thomas MB, et al Impact of daily temperature fl uctuations on dengue virus transmission by Aedes aegypti. Proc Natl Acad Sci U S A [Internet]. 2011 [cited 2012 Jul 5];108(18):1–6. Available from: http://www.pubmedcentral.nih.gov/articlerender.fcgi?artid=3088608&tool=pmcentrez&rendertype=abstract 10.1073/pnas.1101377108 PMC3088608 21502510

[25] Morrison AC, Gray K, Getis A, Astete H, Sihuincha M, Focks D, et al Temporal and geographic patterns of Aedes aegypti (Diptera: Culicidae) production in Iquitos, Peru. J Med Entomol. 2004;41(6):1123–42. 15605653 10.1603/0022-2585-41.6.1123

[26] Hammond SN, Gordon AL, Lugo EDC, Moreno G, Kuan GM, Lopez MM, et al Characterization of Aedes aegypti (Diptera: Culcidae) production sites in urban Nicaragua. J Med Entomol [Internet]. 2007;44:851–60. Available from: <Go to ISI>://WOS:000249179000019 10.1603/0022-2585(2007)44[851:coaadc]2.0.co;2 17915519

[27] Koenraadt CJM, Harrington LC. Flushing effect of rain on container-inhabiting mosquitoes Aedes aegypti and Culex pipiens (Diptera: Culicidae). J Med Entomol. 2008 1;45(1):28–35. 18283939 10.1603/0022-2585(2008)45[28:feoroc]2.0.co;2

[28] Jones CE, Lounibos LP, Marra PP, Kilpatrick a. M. Rainfall Influences Survival of Culex pipiens (Diptera: Culicidae) in a Residential Neighborhood in the Mid-Atlantic United States. J Med Entomol. 2012;49(3):467–73. 22679852 10.1603/me11191 PMC3375620

[29] Koh BKW, Lee CN, Kita Y, Choon ST, Li WA, Kit YW, et al The 2005 dengue epidemic in Singapore: Epidemiology, prevention and control. Ann Acad Med Singapore [Internet]. 2008 7;37(7):538–45. Available from: http://www.ncbi.nlm.nih.gov/pubmed/18695764 18695764

[30] Hii YL, Rocklöv J, Ng N, Tang CS, Pang FY, Sauerborn R. Climate variability and increase in intensity and magnitude of dengue incidence in Singapore. Glob Health Action [Internet]. Co-Action Publishing; 2009 1 [cited 2012 Mar 23];2:1–9. Available from: http://www.pubmedcentral.nih.gov/articlerender.fcgi?artid=2799326&tool=pmcentrez&rendertype=abstract 10.3402/gha.v2i0.2036 PMC2799326 20052380

[31] Ng L-C, Chem Y-k., Koo C, Mudin RNB, Amin FM, Lee K-S, et al 2013 Dengue Outbreaks in Singapore and Malaysia Caused by Different Viral Strains. Am J Trop Med Hyg [Internet]. ASTMH; 2015 [cited 2015 Nov 4];92(6):1150–5. Available from: http://www.ajtmh.org/cgi/doi/10.4269/ajtmh.14-0588 10.4269/ajtmh.14-0588 PMC4458818 25846296

[32] Reiter P. Dengue Control in Singapore. In: Dengue in Singapore [Internet]. 1998. p. 213–42. Available from: http://www.cabdirect.org/abstracts/19990502507.html

[33] Heng B, Goh K, Neo K. Environmental temperature, Aedes aegyptihouse index and rainfall as predictors of annual epidemics of dengue fever and dengue haemorrhagic fever in Singapore. Singapore Minist Environ [Internet]. 1998 [cited 2015 Nov 5];138–49. Available from: http://www.cabdirect.org/abstracts/19990502502.html

[34] Lee KS, Lo S, Tan SSY, Chua R, Tan LK, Xu H, et al Dengue virus surveillance in Singapore reveals high viral diversity through multiple introductions and in situ evolution. Infect Genet Evol [Internet]. Elsevier B.V.; 2012;12(1):77–85. Available from: 10.1016/j.meegid.2011.10.012 22036707

[35] Loo YY, Billa L, Singh A. Effect of climate change on seasonal monsoon in Asia and its impact on the variability of monsoon rainfall in Southeast Asia. Geosci Front [Internet]. 2014 [cited 2015 Nov 4];1–7. Available from: 10.1016/j.gsf.2014.02.009

[36] Xu H-Y, Fu X, Lee LKH, Ma S, Goh KT, Wong J, et al Statistical modeling reveals the effect of absolute humidity on dengue in Singapore. PLoS Negl Trop Dis [Internet]. 2014;8(5):e2805 Available from: http://www.pubmedcentral.nih.gov/articlerender.fcgi?artid=4006725&tool=pmcentrez&rendertype=abstract 10.1371/journal.pntd.0002805 PMC4006725 24786517

[37] Mattingly P. Contributions to the mosquito fauna of Southeast Asia. XII. Illustrated keys to the genera of mosquitoes (Diptera, Culicidae). 1971 [cited 2015 Nov 4]; Available from: http://oai.dtic.mil/oai/oai?verb=getRecord&metadataPrefix=html&identifier=AD0729109

[38] Huang Y-M. A Pictorial Key to the Mosquito Genera of the World: Including Subgenera of Aedes and Ochlerotatus (Diptera: Culicidae). Center for Insect Systematics, Kangwon National University; 2002.

[39] Rueda LM. Pictorial keys for the identification of mosquitoes (Diptera: Culicidae) associated with dengue virus transmission Zootaxa. 2004 1–60 p.

[40] Murrell EG, Damal K, Lounibos LP, Juliano SA. Distributions of competing container mosquitoes depend on detritus types, nutrient ratios, and food availability. Ann Entomol Soc Am. 2011;104(4):688–98. 22707761 10.1603/AN10158 PMC3375989

[41] Focks D a, Alexander N. Multicountry study of Aedes aegypti pupal productivity survey methodology. Trop Med [Internet]. 2007;31:56 Available from: http://www.searo.who.int/LinkFiles/Dengue_Bulletins_cbr4_vol31.pdf

[42] Focks DA, Bangs MJ, Church C, Juffrie MNS. Transmission thresholds and pupal / demographic surveys in Yogyakarta, Indonesia for developing a dengue control strategy based on targeting epidemiologically significant types of water-holding containers. Dengue Bull. 2007;31:83–102.

[43] Barrera R. Simplified pupal surveys of Aedes aegypti (L.) for entomologic surveillance and dengue control. Am J Trop Med Hyg. 2009;81(1):100–7. 19556574

[44] Dobbs R, Stockton G. Playing hide and seek with El Nino. Nat Publ Gr [Internet]. 2015 [cited 2015 Nov 4];5(9):791–5. Available from: 10.1038/nclimate2775

[45] IPCC. Climate Change 2014 Synthesis Report. Contrib Work Groups I, II III to Fifth Assess Rep Intergov Panel Clim Chang. 2014;1–151.

