# Supplementary figures and images for: A Sequence of Flushing and Drying of Breeding Habitats of Aedes aegypti (L.) Prior to the Low Dengue Season in Singapore
Source: PLoS Negl Trop Dis. 2016 Jul 26;10(7):e0004842. doi: 10.1371/journal.pntd.0004842 (PMC4961380; doi:10.1371/journal.pntd.0004842)

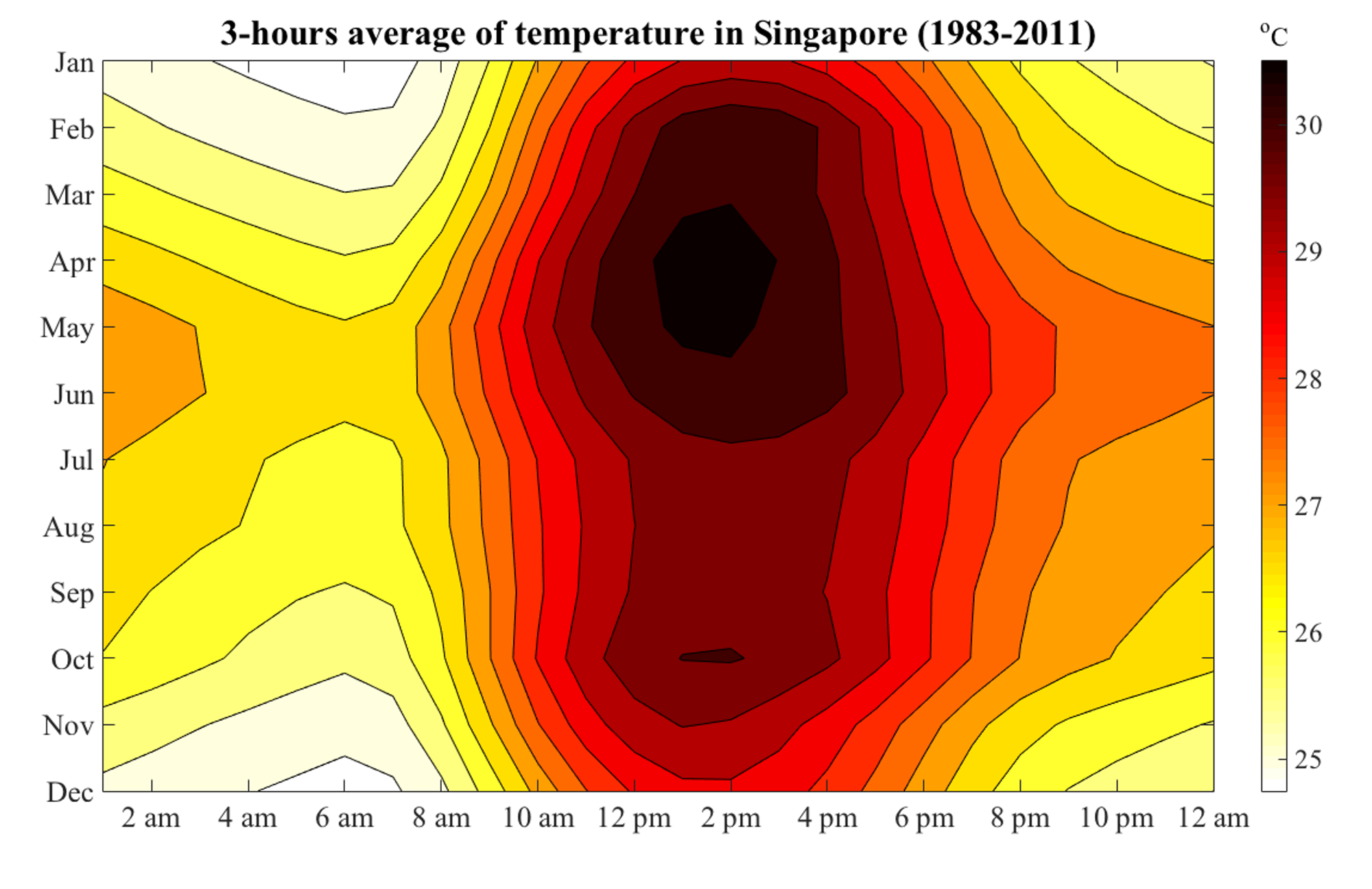

Supplement: S1 Fig — Data source: Changi station– National Environment Agency of Singapore (NEA). (TIF) [file pntd.0004842.s001.tif]
