# Supplementary material for: A Sequence of Flushing and Drying of Breeding Habitats of Aedes aegypti (L.) Prior to the Low Dengue Season in Singapore
Source: PLoS Negl Trop Dis. 2016 Jul 26;10(7):e0004842. doi: 10.1371/journal.pntd.0004842 (PMC4961380; doi:10.1371/journal.pntd.0004842)
Supplement: S1 Table — (DOCX) [file pntd.0004842.s002.docx]

**Supporting Table S2.** Optimization of dengue vector control strategies according to the monsoon period in Singapore

| **Strategy** | **Pre-seasonal (or Low dengue season)** | | **Seasonal (high dengue season)** |
| --- | --- | --- | --- |
| **Targets (measures)** | - Eliminate Indoor breeding (SR/LO/GT) - Adult control where indoor breeding found (IRS/SS). - Monitor outdoor habitats | - Eliminate Indoor breeding (SR/LO/GT) - A preseason treatment of dry drains/roof gutters (CL/BC) - Outdoor discarded receptacles (SR) | - Eliminate Outdoor breeding (SR/CL/BC) - Eliminate Indoor breeding (SR/LO/GT) - Adult control where outdoor breeding found (SS). - Monitor indoor/outdoor habitats |
| **Hydrologic conditions** | Flushing conditions (heavy rainstorms) | Drying conditions | Stagnation conditions |
| **Monsoon** | Early Northeast (NE) monsoon | Late NE monsoon | Southwest (SW) monsoon |
